# Supplementary material for: Surface, Interface, and Temperature Effects on the Phase Separation and Nanoparticle Self Assembly of Bi-Metallic Ni0.5Ag0.5: A Molecular Dynamics Study
Source: Nanomaterials (Basel). 2019 Jul 21;9(7):1040. doi: 10.3390/nano9071040 (PMC6669487; doi:10.3390/nano9071040)
Supplement: Supplementary file 1 [file nanomaterials-09-01040-s001.pdf]

Supplementary Materials

Surface, Interface, and Temperature Effects on the Phase Separation and Nanoparticle Self Assembly of Bi-Metallic Ni0.5Ag0.5: A Molecular Dynamics Study

Ryan H. Allaire [1], Abhijeet Dhakane [2], Reece Emery [3], P. Ganesh [2], Philip D. Rack [2,3], Lou Kondic [1], Linda Cummings [1] and Miguel Fuentes-Cabrera [2,*]

[1] Department of Mathematical Sciences, New Jersey Institute of Technology, Newark, NJ 07102, USA
[2] Center for Nanophase Materials Sciences, Oak Ridge National Laboratory, Oak Ridge, TN 37831, USA
[3] Department of Materials Science and Engineering, The University of Tennessee, Knoxville, TN 37996, USA
* Correspondence: fuentescabma@ornl.gov

S1. Form of EAM Potential

According to Zhou et al., the total energy, E, can be expressed as:

$$E = \sum_i F_i(\rho_i) + \frac{1}{2}\sum_{i,j,i\neq j} \phi_{i,j}(r_{i,j}) \ ,$$

which is composed of an embedding energy, F_i, as a function of the atomic electron density, ρ_i, and a pairwise energy, $\phi_{i,j}$, between atoms i and j which are separated by a distance $r_{i,j}$ [1]. For alloys, it is necessary to incorporate different forms for the pairwise energy depending on if the atoms are of the same or different type. More information regarding the determination of the parameters for the system considered here, Ni-Ag, can be found in [1].

S2. Calibration of Lennard-Jones (LJ) Potential

To calibrate the appropriate value of ϵ, σ, Ni and Ag bulk structures, containing 55296 atoms, at were created at 1900 K and 1750 K, respectively, by sampling from NPT, NVT, and then NVE. They were then placed upon 1 layer of graphene. The well-depth parameter, ϵ, was tuned so that the resultant droplets had wetting angles consistent with the ranges found in literature [2–7] whereas the values of σ were held fixed at 2.8 (Ni) and 3.006 (Ag) angstroms. The wetting angles of the droplets were computed using a distribution of ImageJ software, Fiji, [8] along with the Contact Angle plugin developed by Marco Brugnara, and were averaged over four images (front, back, right, left).

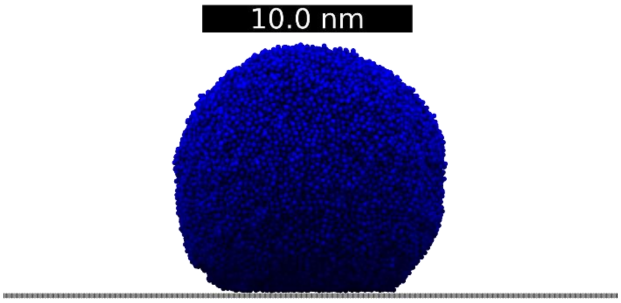

(a)

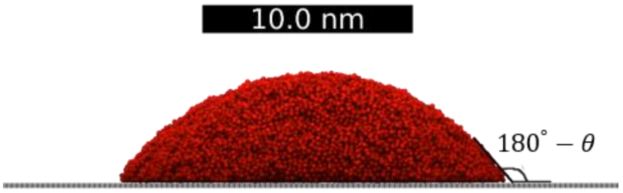

(**b**)

Figure S1. Ag (**a**) at 1750K and Ni (**b**) at 1900 K equilibrated on graphene (grey) using the NVT ensemble on the metal, while holding the graphene fixed. These pictures represent one of four pictures used to calculate the wetting angles.

S3. Complete List of Atomic Concentration Distribution Analysis:

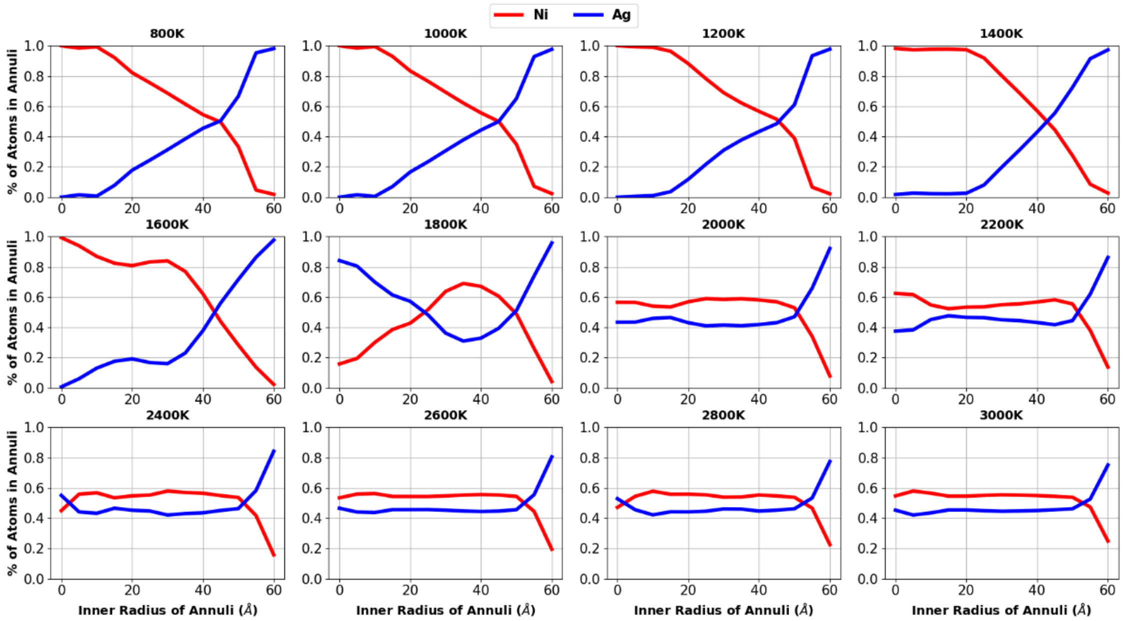

Figure S2. Atomic concentration distribution analysis for the droplets at 200K increments from 800K to 3000K. Color code: Ni, red; Ag, blue.

1. Zhou, X.W.; Johnson, R.A.; Wadley, H.N.G. Misfit-energy-increasing dislocations in vapor-deposited CoFe/NiFe multilayers. *Physical Review B* **2004**, *69*, 144113, doi:10.1103/PhysRevB.69.144113.
2. Bozack, M.J.; Bell, A.E.; Swanson, L.W. Influence of surface segregation on wetting of liquid metal alloys. *The Journal of Physical Chemistry* **1988**, *92*, 3925-3934.
3. Hlinka, J.; Weltsch, Z. Relation between the Wetting Property and Electrical Conduction of Silver-Gold (Ag-Au) Alloys. *periodica polytechnica* **2013**, *41*, 95-98.
4. Lee, J.; Seo, K.; Hirai, N.; Takahira, N.; Tanaka, T. Intrinsic contact angle and contact interaction between liquid silver and solid graphite. *Metals and Materials International* **2007**, *13*, 83-86.
5. Naidich, Y.V.; Perevertailo, V.M.; Nevodnik, G.M. Wetting of graphite by nickel as affected by the liquid-phase dissolution process of carbon. *Soviet Powder Metallurgy and Metal Ceramics* **1971**, *10*, 45-47.
6. Ricci, E.; Novakovic, R. Wetting and surface tension measurements on gold alloys. *Gold Bulletin* **2001**, *34*, 41-49.

7. Weltsch, Z.; Lovas, A.; Takács, J.; Cziráki, Á.; Toth, A.; Kaptay, G. Measurement and modelling of the wettability of graphite by a silver-tin (Ag-Sn) liquid alloy. *Applied Surface Science* **2013**, *268*, 52-60.
8. Schindelin, J.; Arganda-Carreras, I.; Frise, E.; Kaynig, V.; Longair, M.; Pietzsch, T.; Preibisch, S.; Rueden, C.; Saalfeld, S.; Schmid, B., et al. Fiji: an open-source platform for biological-image analysis. *Nature Methods* **2012**, *9*, 676, doi:10.1038/nmeth.2019
